# A Successful Laparotomy at Eighty-one Years

**Published:** 1896-09

**Authors:** 


### A SUCCESSFUL LAPAROTOMY AT EIGHTY-ONE YEARS.

Dr. J. F. Binnie reports a case of gall-bladder disease associated with great pain, in a lady patient eighty-one years old. This is probably a greater age than any other recorded case of this disease. The patient desired relief through operation, and the doctor very skillfully performed a cholecystotomy and fastened the edges of the gall-bladder to the external wound. The patient recovered nicely, but being annoyed by the discharge of bile from this opening, she requested a second operation, which was performed last week, and consisted in establishing an anastomosis between the gall-bladder and the intestine. At the present writing this second operation promises to be a marked success. Can any one send us the record of a successful operation of the kind at a more advanced age?—*Exchange.*

